# A Transcriptomics Approach Reveals Putative Interaction of *Candidatus* Liberibacter Solanacearum with the Endoplasmic Reticulum of Its Psyllid Vector

**DOI:** 10.3390/insects10090279

**Published:** 2019-09-02

**Authors:** Saptarshi Ghosh, Ola Jassar, Svetlana Kontsedalov, Galina Lebedev, Chunxia Wang, Donielle Turner, Amit Levy, Murad Ghanim

**Affiliations:** 1 Department of Entomology, the Volcani Center, Rishon LeZion 7505101, Israel; 2 Citrus Research and Education Center, University of Florida, Lake Alfred, FL 33850, USA; 3 Department of Plant Pathology, University of Florida, Gainesville, FL 32601, USA

**Keywords:** psyllid, bacterial pathogen, insect vector, Liberibacter, ER stress, ERAD, UPR

## Abstract

*Candidatus* Liberibacter solanacerum (CLso), transmitted by *Bactericera trigonica* in a persistent and propagative mode causes carrot yellows disease, inflicting hefty economic losses. Understanding the process of transmission of CLso by psyllids is fundamental to devise sustainable management strategies. Persistent transmission involves critical steps of adhesion, cell invasion, and replication before passage through the midgut barrier. This study uses a transcriptomic approach for the identification of differentially expressed genes with CLso infection in the midguts, adults, and nymphs of *B. trigonica* and their putative involvement in CLso transmission. Several genes related to focal adhesion and cellular invasion were upregulated after CLso infection. Interestingly, genes involved with proper functionality of the endoplasmic reticulum (ER) were upregulated in CLso infected samples. Notably, genes from the endoplasmic reticulum associated degradation (ERAD) and the unfolded protein response (UPR) pathway were overexpressed after CLso infection. Marker genes of the ERAD and UPR pathways were also upregulated in *Diaphorina citri* when infected with *Candidatus* Liberibacter asiaticus (CLas). Upregulation of the ERAD and UPR pathways indicate induction of ER stress by CLso/CLas in their psyllid vector. The role of ER in bacteria–host interactions is well-documented; however, the ER role following pathogenesis of CLso/CLas is unknown and requires further functional validation.

## 1. Introduction

Insect transmitted bacterial diseases, mostly those caused by psyllid-transmitted Liberibacter spp. have been highly invasive in the last decade, rendering hefty economic losses to global crop production. *Candidatus* Liberibacter asiaticus (CLas), causing the citrus greening disease, and *Candidatus* Liberibacter solanacearum (CLso), causing diseases in solanaceous and umbelliferous crops, have particularly caused severe economic losses to the citrus and potato industries [1,2]. CLas is transmitted by the Asian citrus psyllid *Diaphorina citri* Kuwayama (*D. citri*) [3], while CLso is transmitted by specific psyllid species depending upon its haplotype and geographical location. For example, CLso haplotypes infecting solanaceous crops in North America and New Zealand are transmitted by the potato psyllid *Bactericera cockerelli* [4], whereas haplotypes infecting umbelliferous crops like carrots, celery, fennel, and parsley are transmitted by *Trioza apicalis* [5] in Northern Europe and by *Bactericera trigonica* in the Mediterranean region, North Africa, and Middle-East [6,7,8,9,10]. Management practices for Liberibacter-caused diseases primarily revolve around chemical control with pesticides targeting the insect vector [11,12]. Lone reliance on chemical management practices have severely marginalized the returns from crop production [13,14], along with the buildup of insecticide resistance [15]. Understanding the transmission process of Liberibacter by its psyllid vectors, and the molecular mechanisms that underlay this process, could be crucial to devise knowledge-based alternate management practices against Liberibacter-associated plant diseases.

Liberibacter are obligate in nature and require either of their specific plant or psyllid hosts to survive and replicate. Inside the psyllid host, the bacterium follows a circulative, propagative pathway wherein after acquisition by the specific psyllid species, invades the cells of its insect host and propagates within it before being transmitted on to plants. The pathway from acquisition to transmission actualizes with its ingestion through the psyllid’s piercing-sucking mouthparts, followed by passage along the alimentary canal to reach the insect mid/hindgut. The bacteria has then been translocated across the gut lumen into the hemocoel and further enter the primary salivary glands before secretion back into the plant vascular tissues during insect feeding [16]. This circulative path of the bacteria is dependent on several key steps such as focal adhesion, cellular invasion, and cellular trafficking to infect and overcome critical insect tissue barriers such as the midgut and salivary glands [17,18,19]. Moreover, the Liberibacter has to evade the innate immune system of its insect host. Regardless of its importance, mechanisms for establishment, pathogenicity, and dissemination of bacterial plant pathogens inside their insect vectors are mostly unknown [20]. Recently, Liberibacter-containing vacuoles (LCVs) have been closely associated with the endoplasmic reticulum (ER) inside *D. citri* midguts, and these vacuoles have been proposed as its site for replication [19]. The protein biosynthetic and folding functions of the host cell ER and its insulation from host immune responses makes it a favored choice for pathogens as a suitable compartment for its survival and replication [21,22]. However, bacterial proliferation or entry of bacterial toxins inside its host ER physiologically strains the ER compartment, inducing the ER stress response, which triggers cellular stress responses such as the ER associated degradation (ERAD) and unfolded protein response (UPR) to restore ER homeostasis [23]. ERAD is an endogenous quality control system to retro-translocate the misfolded proteins to the cytosol for degradation by the ubiquitin proteasome system. UPR is a cellular reaction to alleviate build-up of unfolded proteins in the ER lumen by reduced protein translation, degradation of misfolded proteins, and also by increased expression of ER chaperones. Prolonged failure in restoration of ER homeostasis further leads to programmed cell death (apoptosis). Host cell ERAD machinery and the UPR both are known to be exploited by pathogens for their advantage [22,23,24,25]. It is unknown how Liberibacter employs the host cell ER for its advantage, and whether disruption of the use of the ER machinery can limit transmission of Liberibacter-caused diseases.

In this work, we used a transcriptomic approach to identify the differentially regulated genes in the psyllid adults, nymphs, and midguts after CLso infection. Differentially expressed candidate genes putatively involved in the focal adhesion and invasion of CLso and psyllid immune response were identified. We provide transcriptomic evidence of the association of the insect host cell ER machinery with CLso, and report upregulation of key genes involved in the ER stress response with CLso infection, indicating induction of ER stress with CLso infection. Comparable to CLso infections in *B. trigonica*, we further investigated whether infection of *D. citri* by CLas also induced ER stress inside the host cells. Relative quantification of the expression of selected marker genes related to ER stress and ERAD when investigated in *D. citri* were also found to be upregulated in CLas infected nymphs of *D. citri*. This study provides the initial identification of important candidate genes putatively involved with Liberibacter transmission by psyllids.

## 2. Materials and Methods

### 2.1. Establishment of Psyllid Colonies and RNA Extraction

Approximately 200 CLso-uninfected adult psyllids were released onto rearing cages with CLso-infected and uninfected celery plants, respectively. Live adult psyllids were removed after 20 days from both CLso-infected and uninfected treatments. Nymphs (4–5 instar) and adults of F1 generation were collected thrice at 10 days intervals from both treatments. Midguts were dissected from a portion of the F1 female adult psyllids collected and stored homogenized in 10 µL aliquots of TRI Reagent (Sigma-Aldrich, Saint Louis, MO, USA). Similarly, the nymphs and female adults were stored homogenized in 50 µL aliquots of TRI reagent. Total RNA was extracted from pooled aliquots of 30 nymphs, 30 adult females, and 70 midguts dissected from adult females, from CLso-infected and uninfected treatments by TRI Reagent. Briefly, the nymphs, adults, and midguts were homogenized in liquid nitrogen with micro-pestles in separate microfuge tubes followed by the addition of 500 µL of TRI Reagent. Phase separation was done by the addition of 0.2 volumes of chloroform and centrifugation at 12,000 × g for 15 min at 4 °C. The upper aqueous phase was removed and mixed with equal volumes of 100% ethanol. RNA was purified using RNeasy mini kit (Qiagen, Hilden, Germany) as per the manufacturer’s protocol. The quality assessment and quantification of the isolated RNA samples were analyzed by a NanoDrop 1000 spectrophotometer (Thermo Fisher Scientific, Waltham, MA, USA). Additionally, a reference RNA sample pool constructed by mixing 2.5 µg of RNA from all of the extracted samples in a separate microfuge tube was sequenced.

### 2.2. Library Construction and Illumina Sequencing and de novo Assembly of the Reference

Library construction, sequencing, assembly, and annotation was done at the Beijing Genomics Institute (BGI, Hong Kong, China). Total RNA of the six samples and the reference pooled sample, after treatment with DNase I, was enriched for mRNA by oligo (dT) beads and fragmented in fragmentation buffer. Second strand cDNA was synthesized using random hexamers, purified, and resolved with EB buffer (10 mM Tris-Cl, pH 8.5) for end preparation and single nucleotide (adenine) addition. The fragments were then connected with paired-end adapters and PCR amplified. Qualitative and quantitative assessment of the library was analyzed using an Agilent 2100 Bioanaylzer (Agilent, Santa Clara, CA, USA) and ABI StepOnePlus Real-Time PCR System (Applied Biosystems, Thermo Fisher Scientific, Waltham, MA, USA), respectively. The library was then sequenced using Illumina HiSeq 4000 (illumina, San Diego, CA, USA).

### 2.3. Denovo Transcriptome Assembly

The raw reads generated were filtered to remove reads with adapter and low quality sequences. Processed reads from the reference pooled sample was used for de novo assembly of the reference transcriptome. De novo assembly of transcript sequences was performed using the Trinity platform [26]. Resulting transcripts were used for gene family clustering with TGICL [27] to obtain final contig consensus sequences. The contigs were annotated by BLAST aligning them to NCBI non-redundant protein (NR, https://www.ncbi.nlm.nih.gov/refseq/about/nonredundantproteins/), NCBI nucleotide (NT, https://blast.ncbi.nlm.nih.gov/Blast.cgi?PAGE_TYPE=BlastSearch), Cluster of orthologous genes (COG, https://www.ncbi.nlm.nih.gov/COG/), Kyoto encyclopedia of genes and genomes (KEGG, https://www.genome.jp/kegg/pathway.html) and SwissProt databases. Gene ontology (GO, https://www.uniprot.org/) and InterPro annotation was obtained by using Blast2Go [28] with NR annotation and InterProScan5 [29], respectively. Segment of contig best mapped to the functional databases as its CDS in a priority order of NR, SwissProt, KEGG, and COG was selected for functional annotation. Contigs that could not be aligned to any database were predicted by ESTScan [30] with BLAST-predicted CDS as the model. These de novo assembled contigs were used as the reference for the gene expression analysis of the samples.

### 2.4. Mapping of Reads and Sample Specific Gene Quantification

The clean reads were mapped to the reference transcriptome using Bowtie2. Quantification of transcripts from mapped reads was done using the RSEM (RNA-seq by Expectation Maximization) tool with normalized expression values as FPKM (fragments per kilobase of transcript per million reads mapped). The expression results were analyzed for their reliability and sample variation by correlation value and PCA analysis between the samples. Differentially expressed genes (DEGs) between samples were screened using the Poisson distribution method. Adjusted P-value (FDR) less than 0.001 and fold-change (log_2_ ratio) greater than 1 was set as the default threshold for significant difference in gene expression. Annotation of gene ontology and pathway enrichment of DEGs was analyzed by GO terms and KEGG pathways. CLso-infected and uninfected samples were screened for DEGs putatively involved in CLso establishment and pathogenesis in *Bactericera trigonica*.

### 2.5. Relative Quantification of Selected DEGs by qRT-PCR

Expression of E3 ligase RNF-185, Derlin-1, and Sel1 in CLso-infected and uninfected midguts was quantified by qRT-PCR analysis. Total RNA was extracted from midguts dissected from 20 CLso-infected and 20 CLso-uninfected adult females by TRI reagent (Sigma Aldrich) with nine replicates each. First strand cDNA was synthesized from 350 ng of total RNA using M-MLV reverse transcriptase (Promega, Madison, WI, USA). Two µL of the diluted cDNA (1:10) was used as the template for q-PCR using gene specific primers (Table 1) and ABsolute blue qPCR SYBR green mix (ThermoFisher Scientific). Specificity of the primers were validated by Sanger sequencing of the amplicons and the calculated efficiency of the primers ranged from 97 to 107%. The genes were normalized to the psyllid elongation factor-1a gene and relative quantities were calculated by the delta-delta Ct method. Significance of the differences of means were analyzed based on one way analysis of variance (ANOVA). Relative expressions of Derlin-1 and IRE1 were also quantified from the *D. citri* nymphs with and without CLas infection. Acquisition of CLas by the nymphal stages of *D. citri* is critical for its transmission and hence only nymph samples were chosen.

## 3. Results

### 3.1. Sequencing of the Reference Transcriptome, De Novo Assembly, and Functional Annotation of Contigs

In order to gain insight into the molecular processes that play a role in CLso transmission, Illumina sequencing of cDNA libraries constructed from the reference pooled RNA from CLso infected or uninfected midguts, nymphs, and adults of *Bactericera trigonica* generated 55 Mb clean reads with a total length of 5.5 Gb bases. De novo assembly with the clean reads using Trinity platform yielded 152,247 transcripts with a total length of 77,708,661 bp, mean length of 510 bp, and N50 value of 716 bp (Table 2). Further clustering of the transcripts with TGICL assembled the reference transcriptome with 57,736 number of contigs, totaling a length of 43,076,597 bp with an average length and N50 value of 746 and 1251 bp, respectively (Table 2). Coding sequences (CDS) predicted with BLAST and ESTscan identified ORFs in 23,501 and 3143 contigs, respectively (Table 2). Functional annotation of the 57,736 contigs against seven databases (NR, NT, SwissProt, KEGG, COG, InterPro and GO) had the highest percentage of contigs (23,567, 40.8%) annotated by NR, with 62.4% of the contigs matching to *Diaphorina citri*.

### 3.2. RNAseq of Samples

**Figure 1:**
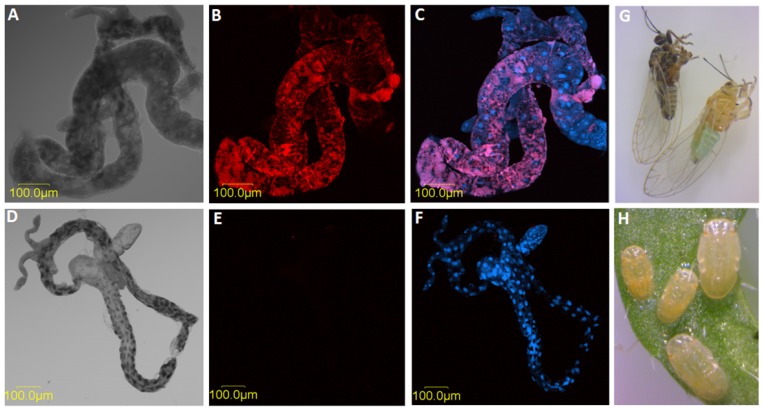
Midgut, nymphs, and adults used for sequencing. (**A**) Dissected midgut from CLso-infected adult psyllid under light microscope, and the same gut that was hybridized with a CLso-specific probe. (**B**) (red) and stained with DAPI (**C**). (**D**–**F)** show a gut dissected from CLso-uninfected psyllid under light microscope (**D**), after hybridization with CLso-specific probe (**E**) and staining with DAPI (blue) (**F**). **G**–**H** show adults (**G**) and nymphs (**H**) of *B. trigonica.*

Illumina sequencing of the six RNA samples extracted from dissected midguts, adults, and nymphs (Figure 1A–H) of CLso-infected and uninfected psyllids generated an average of 12,917,300 clean reads per sample (Table 3). The average mapping ratio to the reference de novo assembled transcriptome was 76.78% by Bowtie2 (Table 3). Sample specific gene expression levels were quantified by RSEM followed by the identification of expressed genes, which averaged 85.29% of the total 57,736 genes in the reference transcriptome. Experiment reliability was tested by correlation statistics, cluster analysis of sample distances, and principal component analysis, which showed low variability among the CLSo infected/uninfected midgut, nymph and adult samples respectively.

### 3.3. Screening Differentially Expressed Genes (DEGs) and Annotation

**Figure 2:**
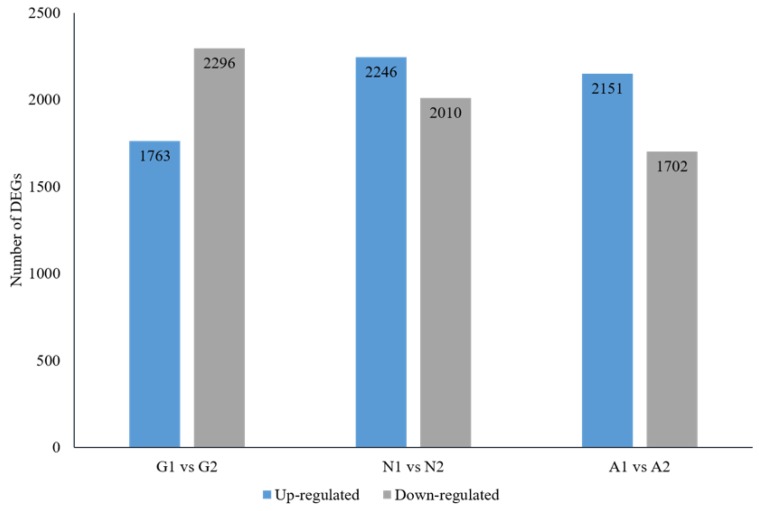
Comparison of the number of differentially expressed genes between CLso-infected and uninfected midguts (G1 vs. G2, respectively), nymphs (N1 vs. N2) and adults (A1 vs. A2).

DEGs between CLso-infected/uninfected midguts, nymphs, and adults of *B. trigonica* were identified by Poisson distribution method followed by its functional annotation. CLso-infected midguts had 1763 and 2296 genes upregulated and downregulated, respectively, compared to the uninfected midguts (Figure 2). CLso-infected nymphs had 2246 and 2010 genes upregulated and downregulated when compared to uninfected nymphs; while 2151 and 1702 genes were upregulated and downregulated in CLso infected adult psyllids when compared to uninfected adults (Figure 2). The DEGs were annotated by GO analysis and grouped to specific gene functions as biological, molecular, and cellular processes by WEGO software (Figure S1A). Biological functions and interactions between DEGs within pathways were analyzed by pathway enrichment analysis based on the KEGG database (Figure S1B).

### 3.4. DEGs of B. Trigonica Putatively Associated with CLso Establishment and Pathogenicity

**Figure 3:**
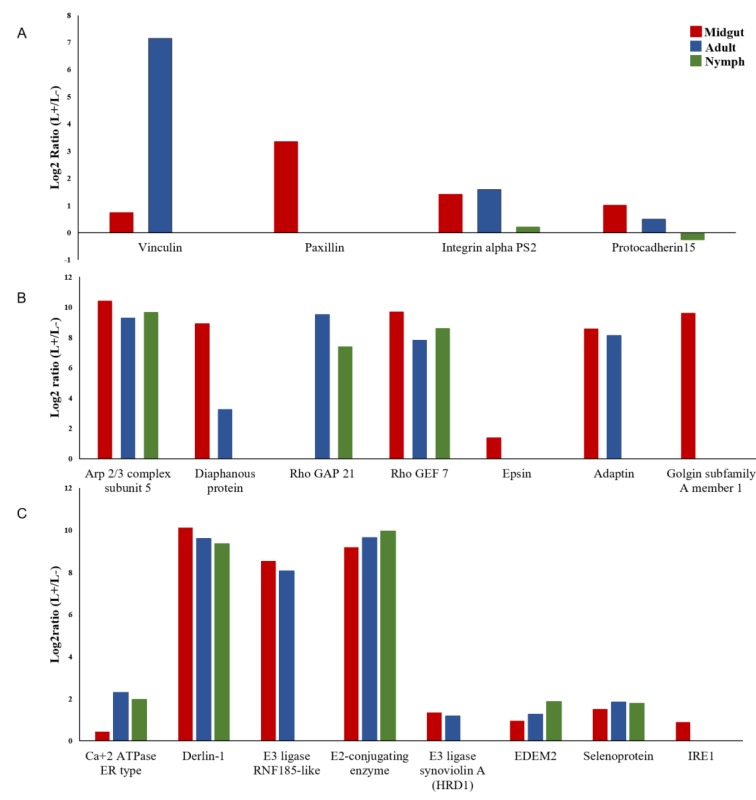
Differentially expressed genes (DEGs) related to CLso pathogenesis in psyllid midguts, nymphs and adults. DEGs related to focal adhesion (**A**), cell invasion and vesicular transport (**B**), and endoplasmic reticulum stress (**C**) are presented. EDEM2 - ER degradation-enhancing alpha-mannosidase-like protein 2; IRE1 - Inositol requiring enzyme 1.

**Figure 4:**
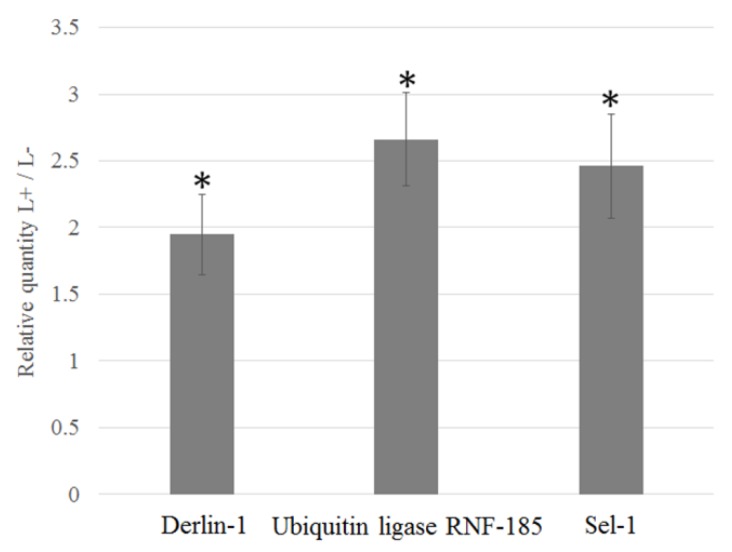
Relative quantification of the ERAD genes Derlin-1, Ubiquitin ligase RNF-185, and Sel-1. The expression is normalized to the elongation factor 1 gene in CLso-infected midguts of *B. trigonica* by qPCR relative to uninfected midguts. Asterisks above columns indicate statistically significant difference when compared to the uninfected control.

Genes known to be involved in pathogen establishment, invasion, and insect immune response were identified and sorted from the differentially expressed contigs. Pathogens interact directly or indirectly with host cell matrix adhesion proteins, leading to cell invasion by rearrangement of actin filaments. Several membrane-cytoskeleton proteins known to be involved in pathogen focal cell adhesion such as vinculin, integrin alpha-8, paxillin, and protocadherin-15 were upregulated in CLso infected midguts (Figure 3A, Table 4). Similarly, multiple genes regulating the actin cytoskeleton such as Actin related protein 2/3 (Arp 2/3) complex, Rho GTPase-activating protein 21 (RhoGAP), Rho guanine nucleotide exchange factor 7 (Rho GEF 7), epsin, diaphanous protein, and adaptin were upregulated in CLso-infected midgut and adult samples (Figure 3B, Table 4). Additionally, disintegrin and metalloproteinase with thrombospondin motif, a gene known to be responsible for pathogen escape across the basal lamina of the insect guts, was upregulated in CLso infected samples (Table 4). Interestingly, several genes involved in proper functionality of the insect host endoplasmic reticulum (ER) were differentially expressed with CLso infection. Signal peptidase complex subunit 1 (SPC1), a gene involved in translocation of proteins into the ER lumen, was upregulated in CLso-infected samples (Table 4). Similarly, calcium-transporting ATPase sarcoplasmic/endoplasmic reticulum type (SERCA), another gene involved in maintenance of calcium homeostasis inside the ER lumen was upregulated after CLso infection (Figure 3C, Table 4). Most interestingly, many key genes involved in the endoplasmic reticulum associated degradation (ERAD) pathway such as Derlin-1, Ring finger protein 185 (E3 ligase RNF-185), Ubiquitin conjugating enzyme, Ubiquitin ligase synoviolin A (Hrd1), ER degradation-enhancing α-mannosidase-like protein-2 (EDEM-2), and Selenoprotein-1 (Sel1) were significantly upregulated (Figure 3C, Table 4). Additionally, Inositol requiring enzyme 1 (IRE1), a key component of the unfolded protein response pathway (UPR) was also upregulated in the CLso-infected midguts (Figure 3C, Table 4). Relative quantification of selected genes from the ERAD pathway such as E3 ligase RNF-185, Derlin-1 and Sel1 by qRT-PCR confirmed their upregulation by 2.74, 1.92, and 2.45 times (*p* value < 0.0001), respectively, in the CLso-infected midguts when compared to the uninfected control midguts (Figure 4). Furthermore, genes involved in host immunity pathways related to autophagy and apoptosis were also differentially regulated (Table 4).

### 3.5. Upregulation of D. Citri Genes Related to ER Stress

**Figure 5:**
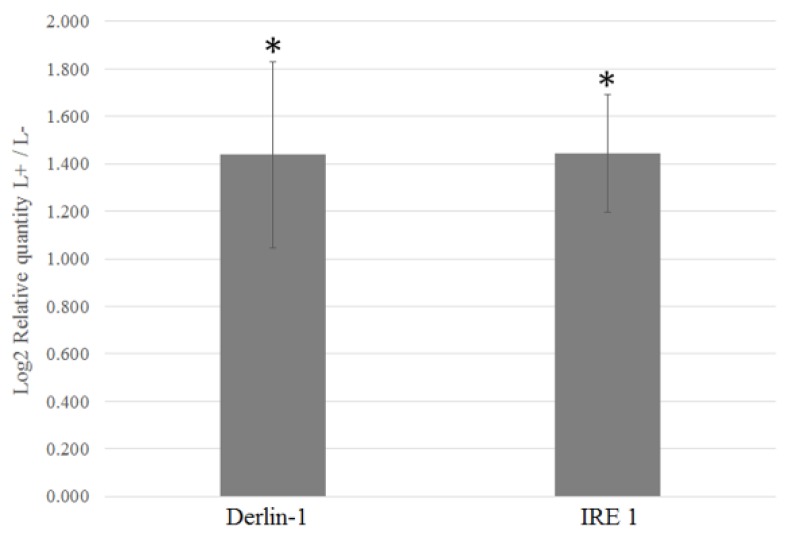
Relative quantification of Derlin-1 and IRE1 genes (Log_2_ expression) in CLas-infected *Diaphorina citri* by qPCR relative to uninfected insects. Asterisks above columns indicate statistically significant difference when compared to the uninfected control.

Previous electron and confocal microscopy studies have demonstrated CLas localization and replication inside Liberibacter containing vacuoles (LCVs) within gut cells and in close association with the endoplasmic reticulum. We therefore hypothesized that ER stress and ERAD may also be involved in CLas transmission. To test this hypothesis, we tested the expression of Inositol-requiring enzyme 1 (IRE1), and degradation in endoplasmic reticulum protein 1 (Derlin-1), which are markers for UPR and ERAD, respectively. Both genes were upregulated in the CLas infected psyllid nymphs when compared to the uninfected nymphs (Figure 5), suggesting a role for these processes in the interaction of both CLso and CLas with their psyllid vectors.

## 4. Discussion

**Figure 6:**
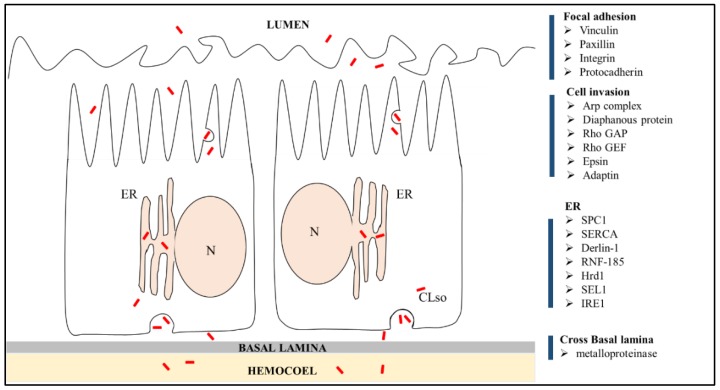
Illustration of putative candidate genes and their involvement in different stages of CLso (red particles) invasion in *B. trigonica* at the midgut/hemocoel interface. N: nucleus; ER: endoplasmic riticulum.

Limited understanding of factors critical for the transmission of Liberibacter by psyllids is a major constraint in the development of alternate management strategies. In this study, the differential expression of genes in psyllid adults, nymphs, and midguts infected and uninfected with CLso were analyzed to identify potential candidate genes involved in the interaction and the transmission of the bacterium (Figure 6). Transmission of pathogens in persistent and propagative mode involves host-pathogen interactions at multiple stages. Persistently transmitted pathogens, immediately after reaching the midgut of the insect, would require successful adhesion to host cells to breach the first physiologically important barrier. In this study, several proteins known to be involved in focal adhesion of bacteria such as vinculin, paxillin, α-integrin, and protocadherin-15 were upregulated in CLso-infected psyllid midguts and adults, indicating their role in Liberibacter adhesion. Focal adhesion proteins are structural protein complexes connecting the cell cytoskeleton to the extracellular matrix, which are often used by pathogenic bacteria for adhesion [32,33]. Differential regulation of vinculin and other adhesion proteins has been previously reported in psyllids with CLas or CLso infections [34,35]. Future investigations on the interactions of these psyllid cell adhesion proteins with CLso/CLas surface proteins are required for further understanding of this mechanism.

Binding of pathogens to extracellular matrix components triggers a cascade of reactions leading to actin polymerization, which drives the invasion and spread of pathogens by endo/exocytosis [32]. Electron micrographs of basal lamina of midguts of psyllids have indicated employment of cytoskeleton rearrangement and endo/exocytosis mechanisms for CLso invasion [17]. The current study showed upregulation of several key genes involved in actin polymerization and cell invasion in CLso-infected midguts such as actin related protein-2/3 sub-complexes, Rho GTPase-activating protein, Rho guanine nucleotide exchange factor, epsin, diaphanous protein, and adaptin. Previous transcriptome profiles of psyllids have also reported differential expression of genes involved in actin polymerization when infected with CLas or CLso [34,35]. Additionally, a disintegrin and metalloproteinase gene with thrombospondin motif was upregulated in the CLso infected samples. Matrix metalloproteinases are known to be involved in re-modeling of the insect gut basal lamina for the escape of pathogens across it [36,37]. The results of this study, along with previous reports, further consolidate the role of actin polymerization in the infection process of Liberibacter inside its psyllid host. Infection and intracellular invasion of host cells by pathogenic bacteria would also trigger immune responses from the psyllid host. In this study, genes known to be involved in the autophagy process such as cathepsin B, cathepsin L, and syntaxin 17 were specifically upregulated in the CLso infected midguts.

Successful invasion of host cells by pathogens is followed by occupying a suitable niche for its replication while avoiding host defenses. The ER of the host cell is often a favored destination for intracellular pathogens [21,22]. CLas/CLso have been reported to associate with the psyllid cell ER to form vacuolar bodies for its replication [19]. This study further highlights the importance of ER in the infection process of Liberibacter inside its insect host cell. Genes involved in normal functioning of the ER such as signal peptidase complex subunit 1 (SPCS1) and calcium-transporting ATPase sarcoplasmic/endoplasmic reticulum type (SERCA) were upregulated in CLso-infected samples. SPCS1 is involved in the removal of signal peptides from nascent proteins for their translocation inside the ER lumen and is known to be utilized by the hepatitis C virus for assembly of infectious virions [38]. SERCA are pumps that are important for maintaining optimum levels of calcium in the ER lumen for protein folding activities of molecular chaperones [39]. Pathogenic infections with the hepatitis C virus [40] and prions [41] are known to induce ER stress by depletion of calcium levels. Thus, upregulation of both SPCS1 and SERCA indicates the interactions between the host cell ER and CLso. Moreover, several key components of the ERAD pathways such as Derlin-1, E3 ligase RNF-185, E2 conjugating enzyme, Hrd1, EDEM-2, and Sel1 were upregulated in CLso-infected midguts and adults. Upregulation of ERAD genes is an indication for ER stress [42,43,44], indicating interactions between the psyllid ERAD machinery and Liberibacter inside its host cell. ERAD involves four major processes: (1) substrate recognition of misfolded proteins for retro-translocation outside the ER lumen; (2) assembly of the translocation channel and retro-translocation of identified misfolded proteins; (3) ubiquitination of translocated ERAD substrates by cytosolic E1 and E2 enzymes; and finally, (4) proteasomal degradation of the misfolded proteins. The upregulated ERAD genes in this study like Derlin-1 [44,45,46], Hrd1 [47], and Sel1 [46,48] are major components of the translocation channel, EDEM-2 functions in ERAD substrate recognition [49], and the ubiquitin ligase RNF185/conjugating enzymes mediate ubiquitylation of ERAD target proteins [50]. Pathogenic viruses and bacteria are known to use this host cell ERAD machinery to their advantage [24,51] to either use it to degrade host immune proteins such as MHC complexes [52,53,54] or use the retro-translocation machinery to escape the ER and enter the cytosol [45,46,55]. Upregulation of Inositol requiring enzyme 1 (IRE1), another key sensor regulating the unfolded protein response (UPR) in CLso-infected midguts, further indicate ER stress following CLso infection. In the current study, we also quantified the relative expression of ER stress response genes in another psyllid species, *Diaphorina citri*, following Liberibacter infection. IRE1 and Derlin-1, two key components of ER stress and ERAD, respectively, were upregulated in CLas-infected *D. citri*, comparable to the CLso-infected *B. trigonica*. Prolonged ER stress eventually leads to apoptosis, another process that has been shown to occur in CLas-infected midguts of *D. citri* [18]. Infection-triggered ER stress and UPR can be beneficial for the pathogen to suppress host cell immune responses or for delivery into the host cell cytosol by retrotranslocation from the ER [25,51,56]. However, ERAD and UPR pathways can also be employed by the hosts to limit pathogen infection and act in the host defense [57,58]. The exact role of ER stress and ERAD in the pathogenesis of CLso/CLas inside the psyllid host is still unknown. Silencing of candidate ERAD/UPR genes identified in this study would be crucial for future understanding of their function in Liberibacter pathogenesis.

This study is the first to associate interactions of a plant pathogen with its insect host ER machinery. The findings of this work could be crucial to understand the infection process of Liberibacter in its psyllid host and finally devise alternative strategies for disease management.

## 5. Conclusions

In the present study, we extended a previous research in which we demonstrated the involvement of the ER in Liberibacter–psyllid interactions. Here, we performed a transcriptomic approach and showed that genes associated with ERAD and UPR mechanisms, which are part of the ER function, are induced upon Liberibacter infection. Those mechanisms are activated upon various stresses including pathogen invasion and disruption of cellular functions such as improper protein folding. The set of genes that we identified suggest an invasion process into the cells and employ adhesion molecules that help invade cellular compartments. Following this invasion, Liberibacter seems to employ the ER machinery to replicate in a safe environment before exiting the gut to the hemolymph and salivary glands, the final step before transmission. Interestingly, the same key genes from the ERAD and UPR responses were induced in both the CLas and CLso systems with the respective psyllid vectors, suggesting that the mechanisms identified are novel and the candidate genes tested in this study could serve, in future functional studies, as targets for disrupting the transmission in both systems.

## Figures and Tables

**Table 1:** Primers used for relative quantification of selected DEGs by qPCR.

Target	Sequence (5’→3’)	Product Size (bp)	Reference	Organism
Derlin 1	F- GGATGGTGTGGCCAGTAA R- CGCACACAGTTCAAAGCA ATG	174	This study	*B. trigonica*
	F- GGTGGTGGTGTCATGGAACT R- CTCAGCTGGTCTTCTTGGCA			*D. citri*
E3 Ligase RNF-185	F- GCAAATACCACCGAACAGAGA R- GACAGGGCCAACAGAATAAGT	147	This study	*B. trigonica*
Sel1	F- ATTGCTCATCTCTTGGCTTGA R- GCAAACTTCTAACCGAGCCT	167	This study	*B. trigonica*
IRE1	F- CTTCACTAGTGTGGCGGAGG R- CCAGCAGCATGAGAGGTGAA			*D. citri*
Elongation factor 1	F- AACATCGTCGTCATTGGACA R- CGCTTGTCAATACCTCCACA	88	[31]	*B. trigonica*
GAPDH	F- GACACTCACTCCTCCATCTTT R- GTATCCGTACTCGTTGTCATACC			*D. citri*

**Table 2:** Summary of the de novo assembly and annotation metrics of the transcriptome library of *B. trigonica*.

Parameter	Total Number	Total Length (bp)	Mean Length (bp)	N50	N70	N90	GC%
Assembled transcripts	152,247	77,708,661	510	716	372	218	40.54
Assembled contigs	57,736	43,076,597	746	1251	634	290	41.08
Predicted CDS-BLAST	23,501	17,708,597	753	1131	696	327	46.68%
Predicted CDS-ESTScan	3143	1,063,341	338	339	264	216	41.61%
NR annotated	23,567 (40.82%)						
NT annotated	14,804 (25.64%)						
SwissProt annotated	16,071 (27.84%)						
KEGG annotated	16,333 (28.29%)						
COG annotated	7034 (12.18%)						
InterPro annotated	12,869 (22.29%)						
GO ontology	2093 (3.63%)						

**Table 3:** Quality and alignment metrics of the RNAseq data of CLso infected and uninfected *B. trigonica* samples.

Sample	Raw Data Size (bp)	Raw Read Number	Clean Data Size (bp)	Clean Reads Number	Clean Data (%)	Q30 (%)	Total Mapped Reads to Reference (%)
G1	639,180,108	13,044,492	632,119,355	12,900,395	98.89	96.9	75.96
G2	639,167,172	13,044,228	633,795,057	12,934,593	99.15	97.0	78.76
N1	639,165,261	13,044,189	633,472,294	12,928,006	99.10	96.8	71.32
N2	639,191,770	13,044,730	633,688,629	12,932,421	99.13	97.0	74.61
A1	639,193,240	13,044,760	631,462,069	12,886,981	98.79	97.1	80.12
A2	639,161,439	13,044,111	633,148,796	12,921,404	99.05	97.1	79.91

**Table 4:** Differentially expressed genes (FDR ≤ 0.001) between CLso-infected and uninfected midguts, adults, and nymphs related to CLso pathogenesis.

Function	Contig ID	Description	Log_2_ratio (L+/L-)		
			Midgut	Adult	Nymph
**Focal adhesion**	CL3226.Contig3	Vinculin	0.74 *	7.15	-
	CL3426.Contig1	Paxillin	3.34	-	-
	Unigene6272	Integrin alpha PS2	1.41	1.58	0.21 *
	CL6794.Contig1	Protocadherin 15-like	1.01	0.50*	−0.26 *
**Cell invasion**	CL3523.Contig2	Arp 2/3 complex subunit 2	-	7.57	-
	CL1981.Contig3	Arp 2/3 complex subunit 5	10.40	9.29	9.66
	CL112.Contig	Formin binding protein 4	8.89	3.23	-
	CL5016.Contig2/3	Rho GTPase-activating protein 21	-	9.50	7.39
	Unigene9133	Actin-binding Rho-activating protein-like	-	2.02	-
	CL3896.Contig2	Rho guanine nucleotide exchange factor 7	9.68	7.80	8.58
	CL7101.Contig2	Epsin	1.36	-	-
	CL1283.Contig1	Clathrin adaptor protein/Adaptin	8.55	8.12	-
	Unigene18634	Filamin	-	9.96	-
	CL2699.Contig1	Dynamin	-	1.32	-
	Unigene33000	phosphatidylinositol-binding clathrin assembly protein LAP	0.75	0.86	-
**Basal lamina egress**	CL5995.Contig1	Disintegrin and metalloproteinase with thrombospondin motifs 18	6.59	7.83	6.77
**Vesicular trafficking**	CL121.Contig2	Golgin subfamily A member 1	9.61	-	-
**Autophagy**	CL3340.Contig3	Cathepsin B	2.51	1.39	
	CL6200.Contig3	Cathepsin L-like	0.34 *	-	
	Unigene18908	Microtubule-associated protein 4	-	2.80	
	CL179.Contig2	Syntaxin-17	1.65	9.37	
**Protein targeting to ER**	CL124.Contig2	Signal peptidase complex subunit 1	1.83	1.32	2.55
**ER Ca^+2^ homeostasis**	CL1301.Contig1	Calcium-transporting ATPase sarcoplasmic/endoplasmic reticulum type	0.44 *	2.3	1.98
**ERAD**	CL3833.Contig2	Derlin-1	10.11	9.61	9.36
	CL7212.Contig6	E3 ubiquitin-protein ligase RNF185-like	8.56	8.07	-
	CL1088.Contig1	ubiquitin-conjugating enzyme E2 J1	9.18	9.64	9.96
	CL3991.Contig1	E3 ubiquitin-protein ligase synoviolin A-like (HRD1)	1.34	1.19*	-
	Unigene8036	ER degradation-enhancing alpha-mannosidase-like protein 2 (EDEM2)	0.95 *	1.27*	1.88
	CL5205.Contig1	Selenoprotein (Sel1)	1.52	1.85	1.78
**UPR**	CL1004.Contig2	Inositol requiring enzyme 1 (IRE1)	0.90 *	-	-
**Apoptosis**	CL5765.Contig1	Inhibitor of apoptosis 1-like	−1.56	−4.47	−3.17
**Chaperone**	Unigene15805	Heat shock protein 70A1	−2.84	−2.01	-

* indicates DEGs with FDR ≤ 0.05.

## Supplementary Materials

The following are available online at https://www.mdpi.com/2075-4450/10/9/279/s1, Figure S1: (**A**) GO functional classification of DEGs between CLso infected and uninfected midguts of *B. trigonica*; (**B**) Pathway enrichment statistics of DEGs between CLso infected and uninfected midguts of *B. trigonica*. Accession numbers: The transcriptome de novo assembly generated in this study is available at the DDBJ/EMBL/GenBank database under the BioProject ID: PRJNA561752. Accession number of the raw reads are SRR10019128 and the transcriptome assembly is GHUW0100000000.
