# Supplementary material for: A Transcriptomics Approach Reveals Putative Interaction of Candidatus Liberibacter Solanacearum with the Endoplasmic Reticulum of Its Psyllid Vector
Source: Insects. 2019 Sep 2;10(9):279. doi: 10.3390/insects10090279 (PMC6780682; doi:10.3390/insects10090279)
Supplement: Supplementary file 1 [file insects-10-00279-s001.pdf]

Supplementary Figure S1:

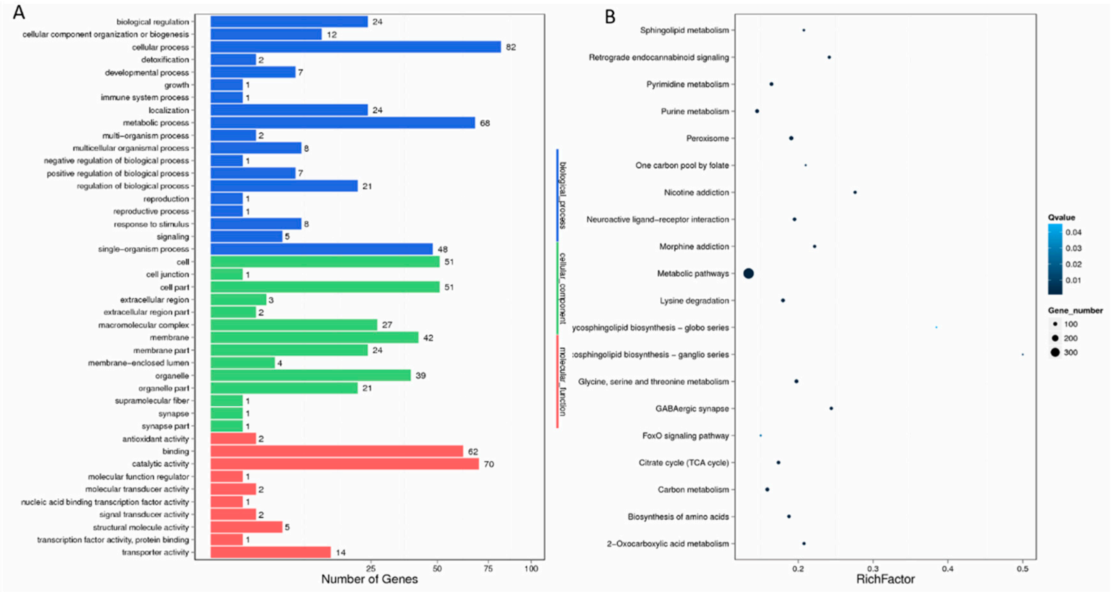

Figure S1. (A). GO functional classification of DEGs beween CLso infected and uninfected midguts of B. trigonica. (**B**). Pathway enrichment statistics of DEGs between CLso infected and uninfected midguts of B. trigonica. Accession numbers: The transcriptome de novo assembly generated in this study is available at the DDBJ/EMBL/GenBank database under the BioProject ID: PRJNA561752. Accession number of the raw reads are SRR10019128 and the transcriptome assembly is GHUW0100000000.
